# Test–Retest Reliability and Minimal Detectable Change of the 6-Minute Step Test and 1-Minute Sit-to-Stand Test in Post-COVID-19 Patients

**DOI:** 10.3390/arm93050033

**Published:** 2025-09-08

**Authors:** Patchareeya Amput, Weerasak Tapanya, Sirima Wongphon, Krittin Naravejsakul, Thanakorn Sritiyot

**Affiliations:** 1 Department of Physical Therapy, School of Allied Health Sciences, University of Phayao, Phayao 56000, Thailand; weerasak.ta@up.ac.th; 2 Department of Traditional Chinese Medicine, School of Public Health, University of Phayao, Phayao 56000, Thailand; sirima.wo@up.ac.th; 3 Division of Urology, Department of Surgery, School of Medicine, University of Phayao, Phayao 56000, Thailand; krittin.na@up.ac.th; 4 Department of Pediatrics, School of Medicine, University of Phayao, Phayao 56000, Thailand; thanakorn.sr@up.ac.th

**Keywords:** 6-minute step test, 6-minute walk test, 1-minute sit to stand test, post-COVID-19, exercise capacity

## Abstract

**Highlights:**

- The 6-minute step test (6MST) and 1-minute sit-to-stand test (1-min-STST) demonstrated excellent test–retest reliability in post-COVID-19 patients, comparable to the 6-minute walk test (6MWT).
- Minimal detectable change (MDC95) values were 5.57% for the 6MWT, 12.21% for the 6MST, and 3.61% for the 1-min-STST, supporting their clinical applicability in monitoring functional improvement.

**What are the main findings?**

The 6MST and 1-min-STST were valid and acceptable for the evaluation of functional capacity in post-COVID-19 patients.

**What is the implication of the main finding?**

Our results confirm that the 6MST and 1-min-STST are useful tools for assessing significant clinical improvements in post-COVID-19 patients.

**Abstract:**

Background: This study aims to determine test–retest reliability and to calculate minimal detectable change (MDC) scores for the functional capacity of the 6-minute step test (6MST) and 1 min sit-to-stand test (1-min-STST), and compare these outcomes with the 6-minute walk test (6MWT) in post-COVID-19 patients. Methods: A total of 42 post-COVID-19 patients aged 18 years or older were recruited for this study. The post-COVID-19 patients were investigated for cardiovascular response parameters induced by a 6MWT, 6MST, and 1-min-STST on two different days, with a five-day interval between the first and second days. Results: The test–retest reliability obtained between the initial measurement and the measurement recorded five days later in the post-COVID-19 patients was excellent for all three of the 6MWT, 6MST, and 1-min-STST. The ICC of the 6MWT was 0.97 with MDC95 at 5.57%. The ICC of the 6MST was 0.93 with MDC95 at 12.21%, while, the ICC of the 1-min-STST was 0.96 with MDC95 at 3.61%. Conclusions: The 6MST and 1-min-STST were valid and acceptable for the evaluation of functional capacity in post- COVID-19 patients and can be used to investigate whether each post-COVID-19 patient had made significant improvement in a clinical setting.

## 1. Introduction

Severe acute respiratory syndrome coronavirus 2 (SARS-CoV-2) causes coronavirus disease (COVID-19) [1], which presents with a wide range of clinical manifestations, primarily affecting the respiratory system. In addition, multiple organs including the heart, kidneys, brain, liver, and muscles can also be affected. Symptoms vary in severity from mild to severe illness [2,3,4]. Even after recovery, some individuals continue to experience persistent symptoms, referred to as post-COVID-19, including dyspnea, fatigue, cough, and weakness [5,6,7]. Fatigue is the most frequently reported symptom, often impacting daily activities [8]. Therefore, evaluating exercise capacity in these individuals is important for designing effective pulmonary rehabilitation programs.

There are various exercise tests used to evaluate exercise capacity levels. Cardiopulmonary exercise testing (CPET) is the gold standard test to investigate aerobic capacity [9]. However, CPET has some limitations for use such as requiring specialized, expensive equipment, and a controlled environment [9]. These limitations might be an obstacle in assessing large populations. Therefore, there are developed field tests which have been developed to evaluate exercise capacity. These are easier to perform and do not require costly equipment. The 6MWT is the gold standard field test used to investigate submaximal levels of exercise capacity [10]. The 6MWT has excellent validity and reliability for assessing exercise capacity in chronic lung diseases [11,12]. However, the 6MWT has technical limitations for use such as the need for a 30 m corridor, which is usually not available in hospitals, rehabilitation centers, and homes [10,13]. Therefore, the 6MST and 1-min-STST tests were developed.

The 6MST is an accessible field test that requires minimal space and demonstrates similar physiological responses to the 6MWT. It has shown strong correlations with the 6MWT in patients with chronic obstructive pulmonary disease (COPD) [14,15] and high validity and reliability in diverse populations [16,17,18,19]. More recently, studies in post-COVID-19 patients have also reported that the 6MST is feasible and useful for identifying reduced exercise tolerance and functional impairment [20,21].

The 1-min-STST is another simple field test, primarily reflecting lower limb muscle strength and endurance [22]. It shows strong correlation with the 6MWT in COPD patients [22] and has been widely used in older adults to evaluate functional status [21]. Importantly, recent investigations suggest that the 1-min-STST may also be sensitive in detecting exercise limitation and fatigue in post-COVID-19 populations [23,24].

However, the information on exercise capacity levels in post-COVID-19 patients using the 6MST and 1-min-STST has not been investigated. Therefore, this study aims to determine test–retest reliability and to calculate minimal detectable change (MDC) scores for the exercise capacity levels of the 6MST and 1-min-STST, and to compare these outcomes with the 6MWT in post-COVID-19 patients.

## 2. Materials and Methods

### 2.1. Study Design

An observational, descriptive, cross-sectional study design was used to investigate test–retest reliability and to calculate MDC scores for the exercise capacity levels of the 6MST and 1-min-STST in post-COVID-19 patients.

### 2.2. Participants

A total of 42 post-COVID-19 patients aged 18 years or older were recruited for this study. The sample size was calculated based on an estimated intraclass correlation coefficient (ICC) [25]. A minimum of 40 participants was required to achieve an expected reliability of 0.80, with a minimum acceptable ICC of 0.60, 90% power, and a 5% significance level. Considering potential dropouts, a 5% attrition rate was assumed. The participants were 18 years old or above, and had a history of COVID-19 infection less than 3 months before the assessment procedure. The post-COVID-19 patients who had a history of cardiopulmonary diseases, musculoskeletal diseases, or neurological diseases that could interfere with the performance during tests were excluded. All post-COVID-19 patients provided written informed consent after being apprised of the study’s protocol. This study was approved by the Clinical Research Ethics Committee of the University of Phayao, Thailand (HREC-UP-HSST 1.3/008/67, 29 February 2024).

### 2.3. Procedure

All measurements were conducted by the researchers over two days, with a five-day interval between the sessions. 1. First day: Participants underwent baseline assessment including demographic and anthropometric data (sex, age, height, weight, BMI). They then performed one 6MWT, one 6MST, and one 1-min-STST. The order of these three tests was randomized using sealed opaque envelopes to minimize order effects. A 30-min rest interval was provided between tests.

2. Second day: Participants repeated the same tests in the same order as Day 1 to ensure consistency.

Before starting to perform the 6MWT, the post-COVID-19 patients had their cardiovascular parameters measured and recorded including heart rate (HR), systolic blood pressure (SBP), diastolic blood pressure (DBP), pulse oxygen saturation (O_2_ sat), rate of perceived exertion (RPE), and leg fatigue. After that, the post-COVID-19 patients were instructed to walk as far as possible for 6 min without running during the test within the corridor space of 30 m. The distance completed in each 6MWT was recorded [10]. In addition, cardiovascular parameters were measured and recorded within 1 minute of performing this test.

Before starting to perform the 6MST, the post-COVID-19 patients had their cardiovascular parameters measured and recorded including HR, SBP, DBP, O_2_sat, RPE, and leg fatigue. After that, the post-COVID-19 patients were instructed to step up and down on a 20 **cm**-high bench as many times as possible in 6 **min**. The score of the test was the total number of steps that could be performed by the subject [19]. Furthermore, cardiovascular parameters were measured and recorded within 1 **min** of performing this test.

Before starting to perform the 1-min-STST, Cardiovascular parameters, including HR, SBP, DBP, O_2_ saturation, RPE, and leg fatigue, were measured and recorded. Patients were then seated with their back straight, arms crossed over the chest, and feet shoulder-width apart slightly behind the knees. After performing two practice repetitions, they completed as many full sit-to-stand movements as possible in 1 min [26]. Cardiovascular parameters were recorded again within 1 min after the test.

### 2.4. Statistical Analysis

Continuous variables were expressed as means and standard deviations (SD). Comparisons between the 6MWT, 6MST, and 1-min-STST were conducted using repeated-measure ANOVA to account for the dependent nature of the data and the group × time interaction. The data of test–retest reliability of the 6MWT, 6MST, and 1-min-STST were assessed using the intraclass correlation coefficient (ICC). The ICC was calculated using a two-way random-effects model with absolute agreement (ICC [2,1]) to assess test–retest reliability. The reliability was established according to the following classification: excellent reliability (ICC ≥ 0.90), good reliability (0.90 > ICC ≥ 0.70), fair reliability (0.70 > ICC ≥ 0.40), and poor reliability (ICC < 0.40). Data distribution was tested for normality using the Shapiro–Wilk test prior to calculating SEM and performing Bland–Altman analysis. The precision of the reliability results was measured by Standard Error of Measurement (SEM), which was calculated using the following equation: SEM = sd × √((1 − r)). The MDC95 was calculated as SEM × 1.96 × √2. Bland–Altman graphs were used to assess measurement agreement. Statistical analyses were performed using IBM SPSS Statistics version 26, with significance set at *p* < 0.05.

## 3. Results

The demographic characteristics of the 42 post-COVID-19 participants are summarized in Table 1. The sample included 20 males (47.62%) and 22 females (52.38%), with a mean age of 54.62 ± 6.90 years. The mean weight was 54.74 ± 5.89 kg, mean height was 1.62 ± 0.06 m, and the mean BMI was 20.87 ± 2.02 kg/m^2^.

The cardiovascular response parameters including HR, SBP, DBP, O_2_ sat, RPE, and leg fatigue were not significantly different between measurement 1 and measurement 2 in the 6MWT, 6MST, and 1-min-STST for the post-COVID-19 patients. The results are shown in Table 2.

The test–retest reliability obtained between the initial measurement and the measurement recorded five days later in the post-COVID-19 patients was excellent for all three of the 6MWT, 6MST, and 1-min-STST. The ICC of the 6MWT was 0.97 with MDC95 at 5.57%. The ICC of the 6MST was 0.93 with MDC95 at 12.21%, while the ICC of the 1-min-STST was 0.96 with MDC95 at 3.61%. Table 3 shows descriptive statistics, ICC, and 95% CI associated, SEM, and MDC95 for the concordance between trials.

**Figure 1:**
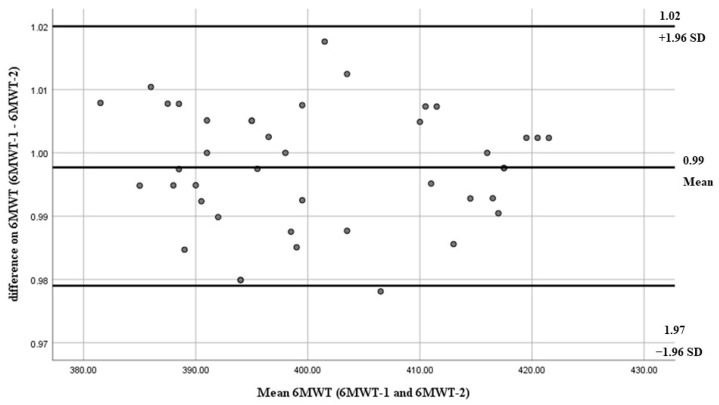
Bland–Altman plots of inter-rater reliability of the 6MWT. The dots represent individual differences between trials, and the gray solid lines indicate the mean difference and the limits of agreement. The x-axis presents the mean score and the y-axis presents the difference between trials.

**Figure 2:**
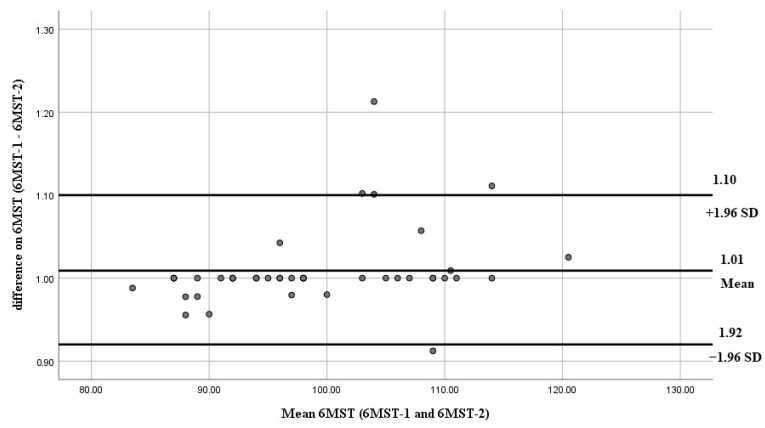
Bland–Altman plots of inter-rater reliability of the 6MST. The dots represent individual differences between trials, and the gray solid lines indicate the mean difference and the limits of agreement. The x-axis presents the mean score and the y-axis presents the difference between trials.

**Figure 3:**
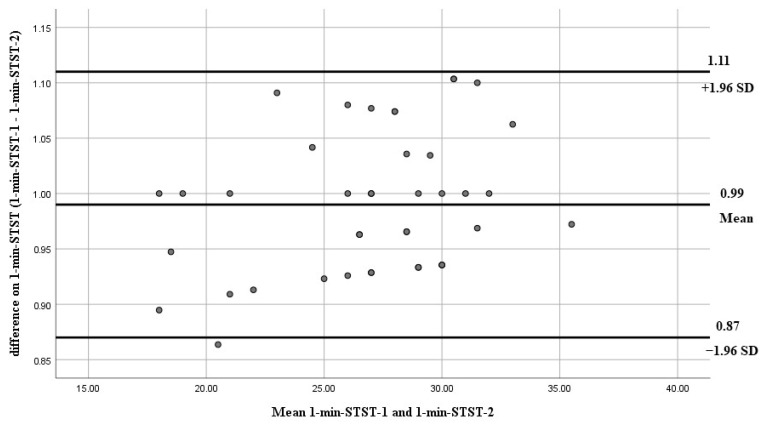
Bland–Altman plots of inter-rater reliability of the 1-min-STST. The dots represent individual differences between trials, and the gray solid lines indicate the mean difference and the limits of agreement. The x-axis presents the mean score and the y-axis presents the difference between trials.

Bland–Altman plots of inter-rater reliability of the 6MWT, 6MST, and 1-min-STST are shown in Figure 1, Figure 2, and Figure 3, respectively. The x-axis presents the mean score, and the y-axis presents the difference between trials.

## 4. Discussion

This current study is the first that investigates the test–retest reliability and calculates the MDC95 scores for the cardiovascular response parameters of the 6MST and 1-min-STST, and to compare these outcomes with the 6MWT in post-COVID-19 patients. Our results showed that the 6MWT, 6MST, and 1-min-STST had excellent test–retest reliability in post-COVID-19 patients (ICC = 0.97, 0.93, 0.96, respectively). Moreover, there were no differences in the cardiovascular response parameters including HR, SBP, DBP, O_2_ sat, RPE, and leg fatigue between the two trials of the three tests in post-COVID-19 patients. These responses may be due to the learning effect.

Our study found that the 6MST showed excellent test–retest reliability in post-COVID-19 patients. This result is consistent with previous studies, which reported that the 6MST showed excellent test–retest reliability in coronary artery disease (CAD) patients (ICC = 0.967), concluding that the 6MST was a reliable test to evaluate functional capacity in CAD patients [27]. In addition, Arcuri JF et al. determined the reliability and validity of the 6MST in healthy participants. Their results showed that the 6MST was a reliable and valid test to investigate exercise tests. Moreover, they suggested that the 6MST is an easy test to perform, can be used in a limited space, and allows better monitoring of the participants [16]. Moreover, the 6MST provided reliable and reproducible estimates of exercise capacity in interstitial lung disease patients [28]. In addition, there is a previous report stating that acute post-COVID-19 patients had no difference in cardiovascular response parameters after performing the 6MST and 6MWT. Their results also showed that the step in the 6MST was correlated with the distance of the 6MWT. These results help to suggest that the 6MST can be used to determine functional capacity in post-COVID-19 patients [29]. Therefore, our results indicated that the 6MST was valid and acceptable for the evaluation of functional capacity in post-COVID-19 patients.

This study showed that the 1-min-STST had excellent test–retest reliability in post-COVID-19 patients. Currently, few studies report the test–retest reliability of the 1-min-STST in post-COVID-19 patients. A previous study reported that the 1-min-STST was a repeatable test without differences between the first and second attempts in post-COVID-19 patients. Furthermore, their results found that the 1-min-STST showed excellent test–retest reliability in post-COVID-19 patients (ICC = 0.984). However, that study did not assess physiological variables during performance of the 1-min-STST [30]. Moreover, a previous study assessed test–retest reliability for the Chester Step Test and 1-min-STST in long COVID-19 patients. Their results found that the 1-min-STST had excellent test–retest reliability in long COVID-19 patients (ICC = 0.98), and was even better than that of the Chester Step Test. It was suggested that the 1-min-STST is a reproducible and reliable instrument to investigate exercise tolerance in long COVID-19 patients [31]. Moreover, a previous study mentioned that the 1-min-STST is a test used to evaluate functional capacity in respiratory chronic diseases and could be performed in low-resource settings [32]. Several studies have reported that the 6MWT is the most widely used test used to assess functional capacity in chronic respiratory diseases. However, our study helps to suggest that 1-min-STST could investigate functional capacity in post-COVID-19 patients.

The 6MST and 1-min-STST assess both cardiorespiratory and muscular function, providing a practical way to evaluate exercise tolerance and pulmonary rehabilitation progress. The 6MST requires repeated stepping, which increases heart rate and oxygen consumption, while the 1-min-STST evaluates lower limb muscle endurance and its contribution to functional capacity. These mechanisms reflect the ability of post-COVID-19 patients to perform daily activities and respond to rehabilitation programs. Compared to previous studies in patients with CAD, interstitial lung disease, and long COVID, our findings confirm that both tests are reliable and valid for assessing functional capacity in post-COVID-19 patients.

The MDC is the smallest measure of change that can be objectified and cannot be attributed to an evaluation error [33]. Furthermore, MDC can be regarded as the threshold to identify a statistically significant change in each individual. This information suggested that clinicians and researchers can use the MDC to determine whether each individual has made significant improvement in a clinical setting. The change score of MDC in each individual between two successive measurements can be viewed as a change with statistical significance [34]. This study also assesses the MDC of the 6MWT, 6MST, and 1-min-STST in post-COVID-19 patients. It was observed that the MDC95 was 5.57% for the 6MWT, 12.21% for the 6MST, and 3.61% for the 1-min-STST. These results suggested that the 6MWT, 6MST, and 1-min-STST had excellent MDC in post-COVID-19 patients [35]. Additionally, there are no reports of the MDC of the 6MST. Therefore, our results are impossible to compare with those previous reports. However, we believe that the MDC of the 6MST in our results can be useful in investigating the effectiveness of interventions applied to post-COVID-19 patients. Currently, there is a study reporting on the MDC of the 1-min-STST in long COVID-19 patients. Our findings indicate the MDC of the 1-min-STST was 12%. The MDC observed in that study could be very useful for the investigation of the effectiveness of interventions applied to these populations [31]. Therefore, our results help to suggest that the 1-min-STST can be useful in determining the effectiveness of investigations applied to post-COVID-19 patients.

Our findings suggest that the 6MST and 1-min-STST are appropriate, low-resource, and reliable functional tests for evaluating exercise capacity in post-COVID-19 patients. They can provide useful information to guide pulmonary rehabilitation programs and monitor individual progress using MDC values.

### Limitations of This Study

This study has several limitations. The sample was relatively small (n = 42), restricted to post-COVID-19 patients without comorbidities, and had a narrow age range (40–66 years), which limits generalizability. All participants had mild COVID-19 symptoms and did not require hospitalization, so the findings may not be applicable to patients with moderate or severe disease. Vaccination status was recorded, but other factors such as pre- and post-infection physical activity levels were not assessed. Although the 6MWT, 6MST, and 1-min-STST showed excellent reliability, gas exchange measurements, autonomic function tests, and deeper analyses of MDC differences were not conducted. Subgroup analyses by sex or age were also not performed. Future studies with larger and more diverse cohorts, including younger participants, those with more severe disease, and individuals with common comorbidities, are warranted to strengthen the external validity and applicability of these functional assessments in a more representative post-COVID-19 population.

## 5. Conclusions

The 6MST and 1-min-STST were valid and acceptable for the evaluation of functional capacity in post-COVID-19 patients and can be used to investigate whether each post-COVID-19 patient had made significant improvement in a clinical setting.

## Figures and Tables

**Table 1:** Characteristics of the post-COVID-19 patients.

Variables	Mean ± SD	(95% CI) Min–Max
Gender: Male (n, %) Female (n, %)	20 (47.62) 22 (52.38)	
Age (years)	54.62 ± 6.90	(52.47 to 56.77) 40–66
Weight (kg)	54.74 ± 5.89	(52.90 to 56.57) 45–68
High (m)	1.62 ± 0.06	(1.60 to 1.64) 1.52–1.76
BMI (kg/m^2^)	20.87 ± 2.02	(20.24 to 21.50) 16.30–25.39

Denote: n = number; kg = kilograms; m = meters; BMI = body mass index.

**Table 2:** Cardiovascular response parameters of the post-COVID-19 patients.

6MWT				6MST			1-min-STST		
	Trail 1	Trail 2	*p*-Value	Trail 1	Trail 2	*p*-Value	Trail 1	Trail 2	*p*-Value
Original is left HR (bpm)	88.64 ± 7.70	88.69 ± 6.91	0.915	92.90 ± 6.48	92.88 ± 5.97	0.955	92.40 ± 5.99	91.90 ± 6.17	0.166
SBP (mmHg)	141.14 ± 6.35	140.19 ± 4.79	0.111	144.24 ± 4.55	143.64 ± 4.51	0.263	142.52 ± 3.96	141.88 ± 4.25	0.261
DBP (mmHg)	79.74 ± 7.16	80.31 ± 6.58	0.181	82.57 ± 6.26	82.90 ± 5.64	0.432	83.02 ± 5.63	83.03 ± 5.51	1.000
O_2_ sat (%)	96.64 ± 0.76	96.74 ± 0.63	0.400	96.67 ± 0.52	96.64 ± 0.48	0.710	97.00 ± 0.62	97.02 ± 0.68	0.785
RPE	10.79 ± 1.30	10.95 ± 0.94	0.181	11.26 ± 1.08	11.36 ± 0.91	0.400	9.69 ± 1.22	9.62 ± 1.08	0.628
leg fatigue	1.51 ± 0.61	1.61 ± 0.60	0.400	2.81 ± 0.63	2.64 ± 0.66	0.109	1.93 ± 0.68	1.92 ± 0.75	1.000

Denote: HR = heart rate; SBP = systolic blood pressure; DBP = diastolic blood pressure; O_2_ sat = pulse oxygen saturation; RPE = rate of perceived exertion.

**Table 3:** The test–retest reliability of the 6MWT, 6MST, and 1-min-STST of the post-COVID-19 patients.

**6MWT**				
**Trail 1**	**Trail 2**	**ICC_(3,k)_**	**SEM**	**MDC_95_**
400.60 ± 11.77	401.52 ± 11.88	0.97	4.04	5.57
**6MST**				
**Trail 1**	**Trail 2**	**ICC_(3,k)_**	**SEM**	**MDC_95_**
99.55 ± 10.10	98.62 ± 8.75	0.93	19.41	12.21
**1-min-STST**				
**Trail 1**	**Trail 2**	**ICC_(3,k)_**	**SEM**	**MDC_95_**
26.79 ± 4.55	27.02 ± 4.13	0.96	1.70	3.61

Denote: 6MWT = 6 min walk test; 6MST = 6 min step test; 1-min-STST = 1 min sit to stand test.

## Data Availability

The original contributions presented in this study are included in the article. Further inquiries can be directed to the corresponding author.
